# Antimicrobial Efficacy of Propolis-Containing Varnish in Children: A Randomized and Double-Blind Clinical Trial

**DOI:** 10.1155/2021/5547081

**Published:** 2021-04-26

**Authors:** Edilson Martins Rodrigues Neto, Lídia Audrey Rocha Valadas, Patrícia Leal Dantas Lobo, Said Gonçalves da Cruz Fonseca, Francisco Vagnaldo Fechine, Mara Assef Leitão Lotif, Mary Anne Medeiros Bandeira, Julia Fontinele Mendonça, Karianne Marques de Mendonça, Marta Maria de França Fonteles

**Affiliations:** ^1^Postgraduate Program in Pharmacology, School of Medicine, Federal University of Ceara, Alexandre Baraúna 949, 60430-160, Fortaleza, Brazil; ^2^Postgraduate Program in Development of Medicine, School of Pharmacy, Federal University of Ceara, Alexandre Baraúna 949, 60430-160, Fortaleza, Brazil; ^3^School of Dentistry, Federal University of Ceará, Alexandre Baraúna 949, 60430-160, Fortaleza, Brazil

## Abstract

Dental caries is a sugar-dependent condition common in childhood, which causes microbiological imbalance in dental biofilm. The present study evaluated the antimicrobial efficacy of a 2.5% Brazilian Red Propolis (BRP) dental varnish to prevent caries in children. Seventy-five children with high caries risk, aged between 36 and 71 months and with no caries, were assigned to three groups to receive varnish treatment containing 2.5% BRP, 1% chlorhexidine, or 5% fluoride. The varnish was applied to the occlusal surfaces of the deciduous second molars on the first day of treatment (D1), after 90 days (D90), and 180 days of the start of treatment (D180). Saliva was collected to assess *S. mutans* before each varnish application and 180 days at the end of treatment (D360). Values were expressed in log10 (CFU/mL). Statistics were performed by applying repeated measures of variance analysis, Tukey's multiple comparisons test, and paired *t*-test. In the first dilution (1 : 10), there was microbial load reduction at the following periods: BRP in D0-D90 (*p* < 0.05) and D0-D180 (*p* < 0.01); fluoride in D0-D90 (*p* < 0.001); and chlorhexidine in D0-D180 (*p* < 0.05). In the second dilution (1 : 100), there was microbial load reduction in the groups at the following periods: BRP in D0-D90 (*p* < 0.05) and D0-D180 (*p* < 0.01); fluoride in D0-D180 (*p* < 0.05), and chlorhexidine in D0-180 (*p* < 0.01) and D0-360 (*p* < 0.05). The 2.5% BRP dental varnish was effective in decreasing *S. mutans* colonies in saliva when used within 90 days.

## 1. Introduction

Dental caries is a sugar-dependent condition that can be defined as the process of demineralization of dental enamel and/or dentin induced by acids released by bacteria, presenting multiple factors that can modulate this pathway [1, 2]. Early childhood caries (ECC) can be defined as a condition that affects the deciduous dentition. It is characterized by the presence of at least one decayed tooth (injury with or without cavitation), absence of a tooth (by caries), or the existence of a provisional restoration in a tooth in a child aged 0 to 71 months [3, 4].

Bacterial biofilm consists of a microbiological community organized in an adhesive extracellular matrix that adheres to dental surfaces. Under ideal conditions such as high consumption of fermentable carbohydrates, there may be imbalance in this microsystem, culminating in biochemical and microbiological alterations that favor the demineralization process [5, 6].

In recent years, different formulations have been associated with natural products. Propolis is a resinous complex responsible for sealing bee hives (*Apis mellifera*) and comes from collecting it from different types and different parts of plants. At present, a variety of compounds have been identified in propolis from different geographic samples and botanical diversity. The chemical characterization of brazilian propolis includes prenylated phenolic acids, lignans, terpenes, and terpene alcohols, in addition to p-coumaric acid derivatives [7, 8]. The Brazilian Red Propolis (BRP) differs from other varieties due to the presence of the isoflavonoids vestitol and neovestitoll. [9, 10].

The following can be listed in relation to the pharmacological effects of propolis: antimicrobial activity against bacteria, fungi and viruses, as well as anti-inflammatory, antioxidant, immunomodulatory, and healing activities [7, 11]. Among the chemical constituents present in the BRP, we can mention vestitol and neovestitol, isoflavonoids which demonstrate antimicrobial activity against *Streptococcus mutans (SM)*, *Streptococcus sobrinus*, *Staphylococcus aureus*, and *Actinomyces naeslundii* and anti-inflammatory activity [12].

Dental varnishes are pharmaceutical forms for dentistry use. They are generally composed of a polymer matrix, pharmaceutical excipients, and an active ingredient, usually fluoride, xylitol, or chlorhexidine. The most commonly used polymers are ethylcellulose, chitosan, and acrylate or vinyl acetate, which may be used in the form of polymers alone or in combination [13].

Such formulations have the advantages of high adhesion to the occlusal dental face and the slow and gradual release of the active principles; thus, an extension of the effect of the therapeutic agent conveyed in this pharmaceutical form is either an antimicrobial or anticariogenic agent [14]. In addition, the pharmaceutical form of dental varnish allows a greater contact surface between the active substance and the dental plaque, thus favoring greater interaction between the active substance and the tooth [15, 16].

No BRP dental varnishes were found in intellectual property banks, and the application for a patent of invention under protocol BR1020160190142 was filed. A pilot study was conducted, and the results encouraged further research [17]. The objective of this work was to perform a clinical and microbiological evaluation of 2.5% BRP dental varnish in children.

## 2. Materials and Methods

### 2.1. Ethical Aspects

The study was conducted in accordance with the Declaration of Helsinki, and the protocol was approved by the Research Ethics Committee of the Federal University of Ceara (no. 195,096). Parents were invited to participate in the research and were informed about it and later signed the Written Informed Consent Form (WICF).

### 2.2. Characterization of Propolis Extract

The BRP extract was collected from the city of Marechal Deodoro (9° 44.555′ Latitude South, 35° 52.080′ Latitude West, and altitude of 18.1 m above sea level), a region with a geographical indication granted by the National Institute of Industrial Property, in the state of Alagoas, Brazil. One hundred and fifty grams (150 g) of the red propolis extract was used and dissolved in 1 L of cereal alcohol of greater graduation. A sample of the absolute alcoholic extract was submitted to chemical identification of its constituents at the Pharmacotechnique's Laboratory of the Pharmacy College of the Federal University of Ceara, Brazil, by High-Performance Liquid Chromatography (HPLC) with the main constituents of quercetin, vestitol, and neovestitol being identified. Identification was performed by comparing the chromatographic profile of the BRP samples in relation to the standards of the isolated chemical constituents subjected to the same analysis conditions.

### 2.3. Manipulation of Dental Varnishes

Propolis extract and chlorhexidine were incorporated into the varnishes in the Pharmacotechnical Laboratory of the Pharmacy course of the Federal University of Ceara, Brazil. A commercial fluoride varnish was applied (Fluorniz®-SS White, São Cristovão, Brazil).

### 2.4. Study Design

This is a longitudinal, parallel, randomized, double-blind, controlled clinical trial and adhered to the CONSORT checklist. The clinical phase occurred in the city of Aracati, CE, Brazil, a city where only 0.8% of the population has fluoridated water coverage [18].

Seventy-five caries-free (ICDAS II 0) and healthy children enrolled in public daycare centers were selected to participate in the study. Screening was performed by means of a dental clinic examination performed in a specific clinic by two calibrated professionals (kappa = 0.88). The high-risk caries classification was performed according to the American Academy of Pediatric Dentistry (AAPD) criteria, for example, sugar consumption more than three times a day, lack of access to fluoridated water, presence of visible plaque, poor oral hygiene, and the absence of a professional dentist. The children were divided into 3 groups of 25 participants each. Group I received application of 2.5% BRP varnish (G1), group II received 1% chlorhexidine varnish (G2), and group III received 5% fluoride varnish (G3). Each patient received the varnish application corresponding to their group in the four second deciduous molars. The varnish was applied 1 time for each tooth at three different times: at the first day of treatment (D1), after 90 days (D90), and 180 days after the start of treatment (D180). The presence or absence of caries lesions in the evaluated teeth was also recorded during all clinical evaluations.

Inclusion criteria were children aged 36 to 71 months, the absence of caries, both genders, belonging to public daycare centers, and who had erupted primary molars, as well as the absence of carious lesions (ICDAS II 0). Exclusion criteria were the presence of any systemic disease, use or application of any antibiotic or anti-infective chemotherapy three months prior to the beginning of the study, history of any allergy, or the presence of any active carious lesion.

### 2.5. Saliva Collection

For the clinical trial, the stimulated saliva from each patient was collected at four moments: baseline (D0), after 90 days (D90), 180 days (D180), and 360 days from the start of treatment (D360). Each patient initially chewed a piece of 3 cm plastic film (Parafilm) for 60 s to stimulate the production of saliva and release the bacteria from the dental biofilm. Saliva was collected using a plastic device and stored in sterile microcentrifuge tubes (Eppendorf), which were then stored in a polystyrene box containing ice. All samples were collected in the same session and conditions by the same operator between 9:00 and 11:00 AM to minimize the influence of the circadian rhythms on salivary flow. Thereafter, the varnish was applied by the same operator to the deciduous molars with relative insulation of each patient after Robinson prophylaxis using brushes and pumice. A triple syringe was then used after 10 s to gently dry the varnish.

Each patient received application of the varnish corresponding to their group in the four primary second molars at times D1, D90, and D180. The teeth were professionally cleaned with a Robinson brush and pumice prior to the varnish application. The varnishes were applied with relative insulation in the selected molars using a microbrush. After 10 s, the varnish was subtly air-dried by using the triple syringe. The cotton rolls were removed after 25 s to avoid saliva contamination. The presence or absence of caries was also recorded in all teeth during each evaluation.

### 2.6. Microbiological Analysis

A volume of 0.1 mL of each sample was transferred to a sterile hemolysis tube containing 0.9 mL of saline. This process was repeated twice, establishing dilutions of 1 : 10 and 1 : 100. A volume corresponding to 10 *μ*L of each dilution was seeded in a medium of *Agar mitis salivarius bacitracin* (MSB) in triplicate. The plates were incubated at 37°C for 48 hours in microaerophilic jars and placed in an oven. Colonies with morphological characteristics of *S. mutans* were then counted after this period. Bacteria were expressed as CFU/mL of saliva.

### 2.7. Statistical Analysis

The transformed values of the CFU number were initially analyzed by the Kolmogorov–Smirnov test to verify the normality of the distribution. Thus, mean and standard deviation were calculated for the descriptive statistics, as well as parametric tests were used to analyze the data. Analysis of variance (ANOVA) was used to compare the three groups at each time (intergroup analysis), associated with Tukey's multiple comparison test to verify differences between the paired groups. Comparisons between the different times within each group (intragroup analysis) were performed by repeated measures analysis of variance (ANOVA), associated with the Tukey multiple comparisons test in order to verify differences between paired times. The level of significance was set at 0.05 (5%) in all analyzes, with a *p* value less than 0.05 being considered as statistically significant. GraphPad Prism® software version 5.00 for Windows® (GraphPad Software, San Diego, California, USA, 2007) was used for both statistical procedures and graphing.

## 3. Results

### 3.1. First Dilution 1 : 10

In the first dilution, there was a statistically significant difference in D180 compared to the baseline in the chlorhexidine-treated group (Table 1). A significant difference was observed in the reduction of the number of CFUs of *S. mutans*, in all posttreatment periods with fluoride varnish compared to baseline. Although the fluoride varnish showed a statistically significant reduction only in the first period, this reduction was maintained until the end of the study (Table 1). The BRP varnish group showed a statistically significant reduction of microbial load in the periods of D0-D90 (*p* < 0.05) and D0-D180 (*p* < 0.01) (Table 1).

Therefore, when comparing the reduction of the microbial load generated by the treatment with the varnishes tested, a satisfactory performance of BRP varnish treatment is observed, in the reduction of the CFU of *S. mutans*, especially in the periods intermediates D90 and D180, due to the statistically significant reduction in saliva analysis, at the dilution of 1 : 10.

### 3.2. Second Dilution 1 : 100

In the second dilution, the fluoride varnish showed reduction in microbial load in D180 compared to baseline (*p* < 0.05). In the group treated with the chlorhexidine varnish, there was a significant reduction of CFU in D180 (*p* < 0.01) and D360 (*p* < 0.05) compared to baseline (Table 2).

Table 2 shows, graphically, the evolution of the microbial counts in saliva samples at a dilution of 1 : 100 during the treatment with BRP varnish. There was a decrease in all periods compared to the baseline, but it was only statistically significant in the period D90 (*p* < 0.05) and D180 (*p* < 0.01). In the D360, there was an increase in the CFU analyzed, being statistically significant in relation to D180 (*p* < 0.05).

At the end of the study, the presence of caries was verified in five children treated with the chlorhexidine varnish; in the group treated with BRP varnish, carious lesions were evidenced in a child, although it was not localized in the molars. In the group treated with fluoride varnish, no carious lesions were developed.

## 4. Discussion

In the present study, we evaluated the antimicrobial efficacy of a new varnish containing brazilian red propolis along 360 days and compared with dental varnishes with fluoride and chlorhexidine, in order to prevent dental caries in a high-risk group of children.

Studies in dentistry with natural products especially occur in relation to cariogenic and periodontal biofilm [7, 12–14, 16, 17, 19]. Several studies have validated the use of BRP. An in vitro study which verified the efficacy of 80% alcoholic extract of BRP against *S. mutans* and *S. sobrinus* also verified the ability of the extract to inhibit acid production by microorganisms. This decrease in acid production was attributed to the enzymatic inhibition of cytosolic F-ATPase [20].

Dental varnishes are pharmaceutical forms of dental use that are well accepted by pediatric patients. The advantage is that it is a relatively quick, simple, and safe professional application [16, 17, 21]. The BRP varnish in the present study was able to act inreducing the salivary CFU of *S. mutans*, being effective in the periods of 90 and 180 days after its application. In this context, 2.5% BRP dental varnish applications would demonstrate greater efficacy if they were repeated with a shorter time period. From the data presented in the study, an application every 3 months would be more interesting to include the pharmaceutical product in dental practice.

However, the index of patients who presented dental caries in the group treated with BRP varnish was relatively low, even with the elevation in the CFU analyzed at D360. This fact can be partly explained by the presence of at least two chemical constituents in BRP: vestitol and neovestitol [12, 22] These isoflavonoids have great antioxidant and antimicrobial effects. In an in vitro study, the two chemical constituents were isolated and showed to have the ability to decrease the production of soluble and insoluble extracellular polysaccharides. Thus, the production and adhesiveness of the bacterial biofilm in the dental matrix is decreased, which hinders the cariogenesis process [12]. Another result of the study by Bueno-Silva et al. [12] revealed that these compounds have the capacity to generate stress in the metabolism of *S. mutans*, thereby diminishing the activities of several enzymes, glycosyltransferases being among them, which help in the formation of biofilm, and also of other metabolic routes involved in the production of acid by microorganisms.

An in vitro study evaluated the antimicrobial activity of green propolis varnishes at concentrations of 5, 10, and 15% with chitosan. The varnishes were tested against *S. mutans* microorganisms and other microorganisms being compared with chlorhexidine and nystatin, obtaining superior results [14]. In addition, this study also verified that propolis can be released by the pharmaceutical form of dental varnish for up to nine weeks, releasing 20 to 30% of propolis in the first 24 hours. Therefore, an activity profile similar to that found in the propolis varnish studied in our present study was verified, which was able to present satisfactory results even within 90 and 180 days of the application.

Another in vitro study also evaluated the action of green propolis varnishes at concentrations of 5, 10, and 15% with chitosan against cariogenic microorganisms. *S. mutans*, *S. sanguinis*, *S. salivarius,* and *Lactobacillus casei* microorganisms were tested. Green propolis and chitosan varnishes of different concentrations significantly inhibited the growth of microorganisms, similar to the pure propolis extract, demonstrating that there is no possible incompatibility in the release of propolis from the varnish. In addition, propolis varnishes at all concentrations showed greater inhibition of microbial growth than chlorhexidine [13]. The work of De Luca et al. [13] also conducted a cytotoxicity test of varnishes in different concentrations, wherein it was verified that the green propolis and chitosan varnish showed very low cytotoxicity according to ISO 10993-5 of 2009, which governs the cytotoxicity assays of clinical devices.

James et al. [23] described that the application of chlorhexidine varnish to pediatric patients should occur every 2 to 3 months. In another study, it was verified that the chlorhexidine varnish only had an effect on the prevention of dental caries when applied at intervals of 3 to 4 months [24]. The group treated with chlorhexidine varnish, as expected, showed a statistically significant reduction of the CFU of *S. mutans* in the saliva samples at day 180 at the dilution of 1 : 10 and at days 180 and 360 at the dilution of 1 : 100. However, five children were detected as having dental caries at the end of the study.

There is currently in the literature a weak evidence of using chlorhexidine varnish in the prevention of dental caries. A systematic review evaluated the efficacy of chlorhexidine varnishes in the prevention of dental caries in children, in which it was found that the efficacy of its use is inconclusive [23]. Although chlorhexidine effectively decreases microbial load in the short-term dental biofilm, it is again taken into account that caries is a sugar-dependent disease mediated by multiple factors. Thus, a broader approach is needed to better control this clinical condition [1, 2].

The group treated with fluoride varnish showed a significant reduction of the microbial load in a sustained way during the study on days 90, 180, and 360 when verified in the 1 : 10 dilution. However, a statistically significant reduction of microbial load was only observed at day 180 when counting CFU at the 1 : 100 dilution of saliva. This decrease can be explained by the residual effect of fluoride in the oral cavity and would not specifically present an antimicrobial effect, but it controls the microorganism population in the oral cavity because of the capacity to cause stress in the metabolism of *S. mutans* [25].

## 5. Conclusions

The 2.5% BRP dental varnish was effective in controlling the formation of *S. mutans* colonies in the oral cavity when used within 90 days, thus being a complementary strategy to assist in the control of dental caries.

## Figures and Tables

**Table 1:** Amount of *Streptococcus mutans*, expressed as the logarithm of the number of colony forming units (CFUs) per ml of saliva, measured in saliva samples with a dilution of 1 : 10 on days 0 (pretreatment), 90, 180, and 360 in patients treated with chlorhexidine, fluoride, and propolis varnishes. The data correspond to the mean and standard deviation of the logarithm of the number of CFUs verified in the saliva samples of 25 patients treated with chlorhexidine and fluoride varnish and 24 subjects treated with propolis varnish.

Day	Chlorhexidine	Fluoride	Propolis	Significance (ANOVA)
	Mean ± SD	Mean ± SD	Mean ± SD	
0	0.58 ± 0.43	0.86 ± 0.37^a^	1.01 ± 0.40^c^	*p* = 0.0010
90	0.38 ± 0.23	0.51 ± 0.33^z^	0.64 ± 0.45^a,x^	*p* = 0.0403
180	0.33 ± 0.14^x^	0.41 ± 0.24^z^	0.60 ± 0.40^b,y^	*p* = 0.0042
360	0.55 ± 0.52	0.53 ± 0.44^z^	0.71 ± 0.35	*p* = 0.2869
Significance (repeated measures ANOVA)	*p* = 0.0107	*p* < 0.0001	*p* = 0.0036	

SD: standard deviation; ANOVA: analysis of variance; ^a^(*p* < 0.05), ^b^(*p* < 0.01), and ^c^(*p* < 0.001) denote statistically significant differences in relation to the chlorhexidine varnish on the same day (Tukey's test); ^x^(*p* < 0.05), ^y^(*p* < 0.01), ^z^(*p* < 0.001) indicate statistically significant differences in relation to day 0 in the same group (Tukey's test).

**Table 2:** Amount of *Streptococcus mutans*, expressed as the logarithm of the number of colony forming units (CFUs) per ml of saliva, measured in saliva samples with dilution of 1 : 100 on days 0 (pretreatment), 90, 180, and 360 in patients treated with chlorhexidine, fluoride, and propolis varnishes. The data correspond to the mean and standard deviation of the logarithm of the number of CFUs verified in the saliva samples of 25 patients treated with chlorhexidine and fluoride varnish and 24 subjects treated with propolis varnish.

Day	Chlorhexidine	Fluoride	Propolis	Significance (ANOVA)
	Mean ± SD	Mean ± SD	Mean ± SD	
0	0.45 ± 0.26	0.48 ± 0.20	0.68 ± 0.24^a,c^	*p* = 0.0023
90	0.33 ± 0.13	0.36 ± 0.11	0.45 ± 0.25^x^	*p* = 0.0766
180	0.30 ± 0.00^y^	0.34 ± 0.07^x^	0.40 ± 0.18^a,y^	*p* = 0.0120
360	0.31 ± 0.18^x^	0.40 ± 0.28	0.64 ± 0.36^b,c,z^	*p* = 0.0004
Significance (repeated measures ANOVA)	*p* = 0.0037	*p* = 0.0417	*p* = 0.0021	

SD: standard deviation; ANOVA: analysis of variance; ^a^(*p* < 0.01) and ^b^(*p* < 0.001) denote statistically significant differences in relation to the chlorhexidine varnish on the same day, while ^c^(*p* < 0.05) indicates a statistically significant difference in relation to the fluoride varnish on the same day (Tukey's test); ^x^(*p* < 0.05) and ^y^(*p* < 0.01) designate statistically significant differences in relation to day 0 in the same group, while ^z^(*p* < 0.05) denotes a statistically significant difference in relation to day 180 in the same group (Tukey's test).

## Data Availability

The datasets used in the present study are available from the corresponding author upon reasonable request.
